# Heat Transfer and Fluids Properties of Nanofluids

**DOI:** 10.3390/nano13071182

**Published:** 2023-03-27

**Authors:** S M Sohel Murshed

**Affiliations:** IDMEC, Department of Mechanical Engineering, Instituto Superior Tecnico, University of Lisbon, 1049-001 Lisbon, Portugal; smurshed@tecnico.ulisboa.pt

**Keywords:** nanofluids, hybrid nanofluids, nano/NePCM, thermophysical properties, convective heat transfer, boiling heat transfer features

## Abstract

As it is popular research field, extensive research has been performed in various areas of nanofluids, and most of the studies have demonstrated significant enhancements in their thermophysical properties and thermal transport performance compared to those of conventional thermal fluids. However, there have been unanimous conclusions regarding such enhancements and their underlying mechanisms. Nanofluids’ potential and thermal applications mainly depend on their convective and boiling heat transfer performances, which are also not unbiased in the literature. On top of this, a major challenge with nanofluids is obtaining sustainable stability and persistent properties over a long duration. All these issues are very crucial for nanofluids’ development and applications, and a lot of research in these areas has been conducted in recent years. Thus, this Special Issue, featuring a dozen of high-quality research and reviews on different types of nanofluids and their important topics related to thermophysical and electrical properties as well as convective and boiling heat transfer characteristics, is of great significance for the progress and real-world applications of this new class of fluids.

## 1. Introduction and Summary

Nanofluids have emerged as a hot research field, as evidenced by worldwide research and publications. Despite them being popular and having great potential, the real progress of this field is rather slow, and their real-world application is greatly impeded by numerous complicated challenges, including the unexplored underlying mechanisms of thermophysical and transport properties, a lack of long-term stability, sustainable usefulness, and compatibility with many conventional systems or devices. It was important to launch this Special Issue on the heat transfer and fluids properties and performances of various nanofluids. The aim of this Special Issue was to publish a wide range of topics on nanofluids with special emphasis on thermophysical and heat transfer properties and features, challenges, and applications in order to contribute to the advancement of this field. 

A total of 12 high-quality articles, including two reviews covering wide ranges of important research topics, were published in this Special Issue. Research and key findings, as well as the conclusions of each article, are briefly summarized here.

The first review article [1] in this Special Issue discusses state-of-the-art research on popular carbon-based nanofluids, namely carbon nanotubes (CNT), graphene and nanodiamonds, as well as their applications in thermal and energy systems. It starts with a review of synthesis approaches to carbon-based feedstocks and different fabrication techniques used for nanofluids. The dispersion stability of carbon nanomaterials in base fluids and its effect on the thermophysical properties of nanofluids are detailed. This review also summarizes the development of theoretical models and correlations with the thermophysical properties of nanofluids. Finally, it reviews and assesses the impact of these nanofluids on the performance of various thermal and energy systems, such as parabolic trough solar collectors, nuclear reactor systems, and air conditioning and refrigeration systems.

Research on boiling heat transfer of nanofluids have also attracted interest from researchers, and a lot of research has been conducted on this important thermal topic. It is, therefore, worthwhile to critically review the findings from the literature on this key topic. To this end, Pereira et al. [2] systematically reviewed the possible mechanisms and characteristics of nanoparticle deposition and its impact on various factors, such as surface roughness and wettability, the density of vaporized core points, and thermal resistance. It also attempted to explain the inconsistent data on the boiling heat transfer performance of nanofluids. In addition to highlighting the pros and cons of nanoparticle deposition after extended pool-boiling periods, it also discussed the effect of the nanoparticle layer on long-term thermal boiling features. 

In recent years, hybrid nanofluids have emerged as a popular type of nanofluids. Hybrid nanofluids are currently receiving a lot of attention due to their interesting thermophysical properties and potential. Thus, it is very important to extensively study the stability and important thermophysical and electrical properties of this new type of nanofluid. In this regard, Giwa et al. [3] performed a comprehensive study by characterizing the morphology and stability of dispersed nanoparticles and by measuring viscosity and electrical conductivity of deionized water (DIW)-based multiwalled carbon nanotube (MWCNT)-Fe_2_O_3_ (20:80) nanofluids at different temperatures and concentrations. Based on TEM and UV-Vis analyses, hybrid nanofluid samples were found to be stable and well dispersed. Both the electrical conductivity and viscosity of these nanofluids were augmented with respect to increasing volume concentration. Based on the experimental data, two empirical correlations were proposed for estimating the electrical conductivity and viscosity of hybrid nanofluids. They noticed that the examined hybrid nanofluid possesses less viscosity in comparison with that of the mono-particle nanofluid Fe_2_O_3_/DIW, which suggests that it has good prospects for convection cooling applications.

Zhang and co-workers [4] employed Darcy–Brinkman and energy transport equations in a numerical study of natural convection heat transfer in a porous annulus filled with a cu nanofluid. This study determined the isotherms, streamlines and heat transfer rate under various conditions and parameters, such as Brownian motion, Rayleigh number, nanoparticle volume fraction, nanoparticle size and porosity. The results showed that the volume fraction of nanoparticles has a positive effect on the heat transfer rate, particularly at high Rayleigh and Darcy numbers, and heat transfer is enhanced by the increase in porosity.

The optical properties of nanofluids are crucial for their application in solar energy systems, such as solar collectors, and investigations on the nonlinear optical effects of nanocolloids is of particular interest and importance. In this regard, a numerical study on the appearance of a solitary wave particle concentration in nanofluids under a light field was conducted by Livashvili et al. [5]. In this study, two exact analytical solutions to a nonlinear Burgers–Huxley-type equation were derived and investigated considering the dependence of various parameters such as the coefficients of thermal conductivity, viscosity, and absorption of radiation on the nanoparticle concentration in the nanofluid. An absorption coefficient and light intensity-dependent expression was also obtained for the solitary wave velocity. The concentration wave velocity for the water/silver nanofluid was also numerically determined. 

It is now well established that nanoparticles’ aggregates play a crucial role in the thermal properties and performance of nanofluids. Regarding this important topic, Karagiannakis and co-workers [6] performed a numerical investigation to assess the effects of polydispersity and sintering on the effective thermal conductivity of nanoparticles’ aggregates. This study found a noticeable decrease in the thermal conductivity for elevated polydispersity levels compared to that of aggregates of monodisperse nanoparticles with the same morphological properties. It was also observed that sintered nanoaggregates offer wider conduction paths, and there is a certain monomer sintering degree that offers the largest improvement in terms of heat performance.

In recent years, phase change materials (PCM) have received the attention of researchers from various disciplines particularly due to their great potential in energy storage. In this regard, Ahmed et al. [7] presented a numerical investigation of the impacts of melting on the convective flow of Al_2_O_3_ (nanoparticle)-based PCM within cylindrical tubes containing cross-shape heated sections The results of this study revealed that the flow structures, the irreversibility of the system, and the melting process can be controlled by increasing/decreasing number of the heated fins. Another numerical study on the natural convection within inversed T-shaped enclosure filled by nano-enhanced phase change material (NePCM) was performed by Abderrahmane et al. [8]. Their considered enclosure contains various factors such as a hot trapezoidal fin on the bottom wall, which was saturated with pours media and exposed to a magnetic field. The impacts of the Darcy number, Rayleigh number, nanoparticle concentration and Hartmann number were analyzed. The results showed that heat transfer coefficients (Nu) were substantially affected by the Darcy number (360% increase) and Rayleigh number (740% increase), while the influence of the other parameters was negligible.

In a different study, Zhang et al. [9] performed a molecular dynamics simulation of the behaviors of water nanodroplets impinging on moving (translation and vibration) surfaces. They studied the dynamical characteristics of water nanodroplets under different factors such as Weber numbers, translation velocities, vibration amplitudes and vibration periods of the surface. Their results showed that when the water nanodroplets impinge on translation surfaces, the water molecules not only move along the surfaces, but also rotate around the centroid of the water nanodroplet at the relative sliding stage. An expression for water nanodroplets velocity in the translation direction was also proposed from this study.

The evaluation of the convective heat transfer performance of nanofluids is very crucial for their thermal management applications, particularly as advanced cooling media. In this regard, Afan et al. [10] reported a forced convection study on the thermal and hydraulic performances of nanofluids containing carbon (graphene nanoplatelets (GNPs) and thermally treated graphene (T-Gr)) and two oxides (Al_2_O_3_ and SiO_2_) nanoparticles in pentaethylene glycol (PEG) in a fully developed turbulent flow in a square, heated pipe. The thermal and hydraulic performances of these nanofluids were evaluated in terms of pumping power, performance index (PI), and performance evaluation criteria (PEC). These nanofluids were found to have considerable thermal conductivity improvements (e.g., up to 31.6%, for PEG/GNPs and 29.74%, for PEG/TGr nanofluids at 60 °C). The heat transfer coefficients (*h* and Nu) of these nanofluids were significantly higher than those of the base fluid (PEG). Based on the experimental data, a nonlinear regression was also proposed for a relative pumping power of nanofluids against temperature at different mass fractions.

Phase change-based heat transfer is of great importance not only for nuclear reactor-type cooling, but also for the thermal management of many modern devices and systems that generate a very high heat flux. Besides enhanced thermophysical properties, nanofluids also exhibit significantly enhanced boiling heat transfer performances. An experimental study on the effect of nanoparticle size and concentration on pool boiling heat transfer with TiO_2_ nanofluids on laser-textured copper surfaces was conducted by Hadžić and co-workers [11]. This study aims to explore the possibility of the concomitant enhancement of pool boiling heat transfer. Their results showed a deterioration in the boiling heat transfer coefficient compared to that of pure water (base fluid) on the reference and laser-textured surface, whereas the critical heat flux was substantially improved at 0.1 wt.% concentration of TiO_2_ nanoparticle. They also confirmed that while the surface porosity (laser-induced grooves and microcavities) allowed a notable delay in CHF incipience, the surface superheat was greatly increased.

The final article in this Special Issue by Ajeeb and Murshed [12] reports the thermal performances of distilled water (DW)-based Al_2_O_3_ and TiO_2_ nanofluids in a compact plate heat exchanger (CPHE) by comparing experimental and numerical investigations. A numerical study employing the finite volume method was performed, considering the same CPHE dimensions and operation conditions of the experimental investigation. The results from this study confirmed that the thermal performances of these nanofluids (DW/Al_2_O_3_ and DW/TiO_2_) in CPHE considerably increased by adding nanoparticles to the base fluid (DW). It was concluded that the numerical model is suitable with an acceptable accuracy for the prediction of nanofluids’ performance in CPHE. This study can provide a better understanding of the heat transfer performance and underlying mechanisms of nanofluids in such heat exchange systems, besides highlighting the benefits of using CFD tools for modelling nanofluids with a clarification of their thermal characteristics. 

## 2. Concluding Remarks

This Special Issue aimed to cover various classes of nanofluids, such as conventional nanofluids, hybrid and Nano/NePCM, as well as their important properties and features, such as thermophysical and electrical properties, convective and boiling heat transfer characteristics, from the renowned research groups across the globe. The outcomes of these studies are significant and will contribute greatly towards the progression of this emerging field.

The total number of citations (up to this date) of the published articles in this Special Issue is 117, which indicates the high quality and impact of these articles. The articles in this Special Issue are expected to continue to be useful resources for related industrial professionals and researchers in this field. 
